# Knowledge, Awareness, and Health-Seeking Behaviour regarding Tuberculosis in a Rural District of Khyber Pakhtunkhwa, Pakistan

**DOI:** 10.1155/2020/1850541

**Published:** 2020-04-21

**Authors:** Adeela Khan, Babar Tasneem Shaikh, Mirza Amir Baig

**Affiliations:** ^1^Rights & Health Alliance for Integrated Development, Islamabad, Pakistan; ^2^Health Services Academy, Islamabad, Pakistan; ^3^Field Epidemiology & Laboratory Training Program, National Institute of Health, Islamabad, Pakistan

## Abstract

**Background:**

Pakistan is a country with one of the highest burden of tuberculosis (TB) in the world, and therefore, it is imperative to revisit the design of behaviour change interventions in the program. This study was designed to understand and assess the knowledge, awareness, perceptions, and health-seeking behaviour of general and specifically TB-affected population and to determine the presence and level of stigma and discrimination toward TB patients.

**Methods:**

A mixed-method study was conducted in district Haripur of the Khyber Pakhtunkhwa province, comprising a household survey, whereby 526 individuals were interviewed, and five focus group discussions with various subgroups including TB patients and health workers and authorities. Study sought an ethical approval, and data of all respondents was kept confidential.

**Results:**

Quantitative results show that women were more knowledgeable on symptomatology and spread of TB, and with rising education, awareness on TB improves. The majority of our respondents had the understanding that it is a curable disease, yet some would avoid TB patients. Most of the respondents (both men and women) knew that one must go to a government facility for treatment. Only one-third would speak to doctor first, if they suspect TB-like symptoms. Television was a popular source of information on TB. Qualitative results captured people's perceptions that TB was related with poverty and was still considered a stigma in the community; hence, patients afflicted feared disclosing the disease.

**Conclusion:**

With contextual understanding of communities' knowledge, attitudes, health-seeking behaviour, and care-seeking patterns, it can be concluded that there is a need to increase the awareness about TB symptoms, mode of transmission, prevention, diagnosis and treatment, and destigmatization of the disease through health education.

## 1. Introduction

Tuberculosis is a disease caused by bacteria called *Mycobacterium tuberculosis*. The bacteria usually attack the lungs, but they can also damage other parts of the body. Symptoms include cough for more than 3 weeks, fever with chills, night sweating, loss of appetite, fatigue, and in worst cases blood in sputum with chest pain. TB spreads through the air when a person with TB of the lungs or throat coughs, sneezes, or talks [1]. Pakistan is ranked 5th among 22 countries with the highest burden of tuberculosis (TB) and 27 high multidrug resistant (MDR) TB countries of the world, and it contributes about 60% of the total TB burden in the Eastern Mediterranean Region. Approximately 510,000 new cases occur every year in the country [2]. Evidence suggests that improved awareness regarding TB leads to better treatment outcomes and a better control over the spread of TB [3]. Moreover, studies have shown that increasing patient knowledge about clinical manifestations, transmission, and control methods of TB leads to decreasing annual incidence of TB [4, 5]. Advocacy, Communication and Social Mobilization (ACSM) activities address the key barriers to accessing TB care and completing treatment [6]. The need to keep ACSM planning and implementation a high priority is recognized at all levels because the ambitious goal of TB control will not be met otherwise. To bring about a sustainable social and behavioural change, ACSM interventions need to be evidence-based and culturally adapted [7]. Our study endeavoured to determine the current knowledge and awareness of people of the Khyber Pakhtunkhwa province regarding TB, its treatment and prevention. It examined the current health-seeking practices in a predominantly rural setting where sociocultural and demographic factors are different than the urban setting of the same province. Furthermore, using a qualitative arm of the study, it tried to assess the magnitude of discrimination and stigma faced by TB patients and the determinants of such discriminatory behaviour. The findings of this study could very well inform the program and policy on TB in Pakistan.

In the recent past, a few studies were conducted in order to assess the knowledge, awareness, and health-seeking behaviour of communities regarding TB. However, these were designed to meet specific program objectives. One such survey, conducted in 2011 in 57 districts across Pakistan, mentioned that for implementing behaviour change interventions in specific communities, it is important to learn about their preferred source of information [8]. Another local study identified significant gaps in the awareness and practices of private general practitioners regarding TB diagnosis and treatment [9]. A nationwide survey showed insufficient knowledge of Pakistani population in terms of symptoms, transmission, diagnosis and treatment of TB, and the misconceptions related to spread of TB [10]. TB is a disease of poverty which has a direct effect on health, and hence, the disease further limits the access to healthcare services. It is well documented that level of awareness, knowledge, and perceptions of various health issues are the drivers behind the health-seeking behaviours in any community [11]. On the supply side, the scarcity of budgetary allocations in health system makes it challenging to serve the urban poor and rural communities [12]. In view of the above, there was an obvious need to generate fresh evidence to inform the national TB program to design appropriate strategies and interventions to bring about a positive change in the current situation of ever-increasing TB cases.

This study endeavoured to understand and assess the knowledge, awareness, perceptions, and health-seeking behaviour of general and specifically TB-affected population. It also attempted to determine the presence and level of stigma and discrimination toward TB patients.

## 2. Methods

The scope of study included desk review of the existing literature, preparation of research protocol, data collection tools, pretesting and translation of questionnaires, training of enumerators, data processing (data entry, verification, and analysis), and report writing. The study was conducted in the district Haripur of the Khyber Pakhtunkhwa province in Pakistan, a predominantly rural catchment area. The study used a mixed methods strategy, i.e., both qualitative and quantitative approaches for data collection, as it helped provide information as per objectives of the study. Under the quantitative section, a cross-sectional study was conducted on general public using a validated structured questionnaire. The qualitative section included focused group discussions. The population of the district was taken as the sampling universe.

A Household Survey questionnaire and Focus Group Discussion (FGD) guides for different subgroups and service providers were adapted from the WHO ACSM toolkit [13]. The details of the two components are given in Table 1.

Cultural sensitivities were considered and incorporated during the questionnaire adaptation. Through a written consent form, all the respondents were provided with a detailed overview of the objectives of the study prior to seeking their consent for participation. For minors (below the age of 16), parents were asked to give the consent. Respondents were given the right to refuse participation in the study, to terminate their participation at any time, and to not answer any question/s, which they did not wish to. All interviewers and team members were trained and briefed on ethical issues, including confidentiality. No identifying information (including the use of names and addresses) were recorded in data collection. All participants were identified with the use of a code to allow for the checking of data. All completed questionnaires/guidelines were stored securely, and no access was provided to any irrelevant person during collection, data entry, and analysis. Interviews were held at times and place when respondents were accessible and available (work hours and days). This ensured maximum time utility and minimum refusals of the respondents. The use of same sex interviewers speaking the same language helped to achieve a high response rate.

## 3. Results

Out of 526 sample size, the responses received were 488, thereby achieving a response rate of 92.8%. The respondents included 54% men and 46% women. In terms of age, maximum respondents (84%) were between 15 and 45 years of age. Almost a quarter of them never had any formal schooling, similar proportion attended only primary or secondary school, 42% went to high school, and only a few attended a college-level education. As regards the monthly income, around one-third of the respondents had a monthly household income of less than PkRs10,000 (US$100); 42% earned PkRs10,000-20,000 (US$100-200); 14% reported a monthly income between PkRs20,000-Rs30,000 (US$200-300); and 12% of the respondents reported a monthly income of more than PkRs30,000 (>US$300/month). Any differences in gender, age, and income are discussed only when recorded as significant.

### 3.1. Knowledge about TB, Its Symptoms and Mode of Transmission

Almost all the respondents (99%) reported to have heard about TB. Regarding the knowledge on symptoms of TB, 39% of the respondents mentioned cough, coughing with blood (24%) or coughing with fever (15%), but only 7% mentioned cough for 2-3 weeks (a classical symptoms of TB). Women seem to be more knowledgeable than men (*p* < 0.05) as regards symptoms. Knowledge is more correct with a rising education level, and college/university-educated respondents were more accurate (*p* < 0.05). Around a quarter of them mentioned that it could spread “through the air,” 22% mentioned “coughing or sneezing of TB patients,” and 12% mentioned “eating from the same plate” as the mode of transmission of disease. Around 18% of the respondents did not know the mode of transmission of TB. Again, women were more accurate than men on the mode of transmission (*p* < 0.05), and the correctness of response increased with higher education.

### 3.2. Knowledge about TB Prevention and Its Curability

Regarding prevention of TB, respondents had varied responses: 19% stated that spread of TB could be prevented by covering the mouth during coughing and sneezing, 18% mentioned good nutrition, 13% would avoid eating from the same plate, and 6% will not share the bedding. Around 14% confessed little-to-no knowledge regarding TB prevention. This is significantly associated with gender (men being more informed *p* < 0.05), and those with higher income group and with more education (*p* < 0.05) gave correct answers. Furthermore, an overwhelming majority (96%) of the respondents recognized TB as a curable disease. This was true across gender, age, education status, and household income.

### 3.3. Knowledge about TB Diagnostic and Treatment Services

When asked about the location of TB diagnostic services, about 90% mentioned government hospitals, and no significant difference in responses was seen across gender, age, education, and income level. Regarding knowledge on TB diagnostic tests, 72% reported sputum test, 14% reported blood test, and 8% reported chest X-rays as the diagnostic test of choice, besides few other incorrect responses. When asked about the location of TB treatment services, again, about 90% mentioned government hospitals, and no significant difference in responses was seen across gender, education, and age and income level. Around 74% of the respondents were aware of the fact that TB treatment services are free. No significant difference in responses was noted across gender, age, or income levels, except that 62% with no formal schooling were aware of this fact as compared to 82% with those having a master's level degree.

### 3.4. Perceptions and Attitude regarding the Disease

Around 22% of the respondents perceived TB as a fatal disease, 40% thought it was very serious, whereas 35% were of the view that it was not a serious disease, and others had no idea. As regards the attitude of general public toward the known TB patients, 72% said that there was “no change” in their behaviour or attitude toward the patient, while 5% admitted that they had stopped interacting with the person afflicted with TB. This attitude of avoidance of TB patient seemed to be worsening with increasing education, and the same trend was prevalent across all genders, age groups, and income levels. Enquiring as to how the overall community behaves toward a person suffering from TB, 71% of the respondents said that the community fully supports any TB patient, while 23% said that the patient was avoided. Around 6% shared that the community altogether rejects any interaction with the TB patients.

### 3.5. Health-Seeking Behaviour

Following the diagnosis of the disease, around 45% would visit the nearest government health facility while 41% would seek advice from a private doctor. The rest would consult family for first opinion. More men (64%) as compared to women (36%) would use a government health facility. When asked of the first person to speak if they had TB, around 35% responded they would speak to a doctor or other medical staff, 45% said they would talk to their spouse, and 20% preferred to discuss the issue with their parents. There was gender variation in the responses. More men (73%) said they would talk to their doctor as compared to only 27% women (*p* < 0.05). More women (71%) would talk to their spouse as compared to 29% men (*p* < 0.05).

Besides a household member (40%) or a friend (19%), television was mentioned as the first source of information regarding TB (37%). Only 3% reported doctors as the primary source while only 1% mentioned the community-based lady health workers.

### 3.6. Qualitative Results

The qualitative results show that the knowledge regarding the symptoms and transmission of TB was quite satisfactory among all the participants. Some of the participants even explained the vicious cycle of TB and poverty. Awareness regarding the location of the nearest TB centre was also satisfactory. Most of the participants opined that actually the long duration of therapy lead to noncompliance of the treatment.

*The treatment is very hard, and one has to take it for long period of time. It is the long duration which makes it impossible to continue the medication.* (Teacher)

A direct observation of the treatment by some family elder or *Imam Mosque* was viewed as a useful intervention. However, they complained that the side effects of TB treatment made it very difficult for the patients to perform the daily chores.

*TB does not let you get out of the bed; it is good that I have my daughter to support me throughout my therapy. Otherwise, what would I have done without her.* (Male TB patient)

Disclosing TB status to the family and friends was a serious issue with most of the participants because it is stigmatized and TB patients are discriminated by the larger community. People usually hide their TB status for fear of social exclusion.

*We do not disclose the disease because of community attitude as it creates eventually many difficulties for us to socialize in the larger family.* (Female TB patient).

Healthcare providers reported that TB is regarded as a serious and deadly disease and discussed varying levels of emotions and the perceived stigma in the communities.

*Once I explained a mother about treatment of her daughter and where to get it. She refused to consult the TB centre, saying that she is to marry her daughter in three months and that it is risky to reveal her TB status.* (Lady health worker)

Television, radio, newspapers, and community programs were considered effective sources of TB information. Lady health workers were also mentioned as another reliable source. A lack of IEC materials and the need for more health promotion activities were also emphasized.

*It is the age of electronic media, and we all have access to it. So whatever we learn about the disease, mainly it is from the electronic media.* (Imam mosque)

## 4. Discussion

Haripur is a predominantly rural conservative poor country side district of Khyber Pakhtunkhwa. More than half of the respondents had a monthly income of less than PkRs15,000 per month (US$150) which reflects the socioeconomic profile of the district. This situation calls for addressing poverty through safety nets for health in the province as well as at national level which would go a long way in controlling TB [14]. Our quantitative and qualitative results confirmed that general information about the disease and knowledge of signs and symptoms of TB is quite reasonable and not an issue *per se*, just like those shown in other studies from the developing countries [10, 13, 15]. There are, however, some aspects where women's knowledge was weaker than men, for instance about the disease curability. The knowledge on spread of disease was poor, and this is typical phenomenon noted in other similar settings too [16]. This lack of awareness can result in people being prone to acquire infection. While mentioning how to prevent the disease, no one mentioned BCG vaccination as a method of prevention for TB. This highlights the need for interventions designed to improve knowledge of the community regarding immunization against TB. Although TB was considered to be a serious, life threatening condition, yet respondents were convinced that it was curable, and this finding concurs with earlier studies [17, 18]. Hence, the health-seeking behaviour regarding TB can potentially be improved based on this belief [19]. Diagnosis of TB might lead to stigmatization and associated psychological problems. Therefore, empathetic counselling must be provided to all TB patients. The National TB Control Programme has put considerable efforts in designing a culturally sensitive communication strategy [20].

Social discrimination for fear of disease dissemination is a universal TB-associated stigma [21, 22]. It is a noted fact that people avoid and even reject the interaction with TB patients, leading to their social exclusion and difficulty in finding the marital match [23, 24]. Unfortunately, such attitudes seem to be worsening with an increasing level of education, income, and awareness of TB [8, 20, 25]. Considering the deep rooted character of such beliefs, concentrated and rigorous efforts will be needed such as community awareness and mobilization program in order to challenge this prejudice. Furthermore, it is easier for men to seek advice from doctors, while women have their spouses, friends, or family to talk to as a first resort, which explains the patriarchal cultural norms and societal underpinnings in such study setting [26, 27]. These considerations have to be kept in mind when designing interventions within the TB Control Programme. Since television was reported to be a popular source of information on TB, this should be used for an effective medium of communication for reaching the wider population. However, peer-to-peer counselling and community health workers channel need more attention to communicate with the poorer segments that do not have access to electronic media. Strength of this study is that it is the first study of its kind in the province which has used mixed methods. Nonetheless, this study could have been more robust with more stakeholders involved especially in the qualitative arm.

## 5. Conclusion

Knowledge, attitudes, health-seeking behaviour, care-seeking patterns, and awareness about prevention of TB need to be understood more insightfully in the diverse sociocultural milieu of Pakistan. Health systems' thinking as well as approach, which analyses the sociocultural and politicoeconomic determinants of any disease, must be instigated among the managers and decision-makers en route to address the disease burden in Pakistan. To increase the awareness about TB symptomatology, transmission, prevention, diagnosis, and treatment, focused health education interventions are needed with emphasis on destigmatization of the disease.

## Figures and Tables

**Table 1:** Methodological details of the study.

Methodology	Quantitative component	Qualitative component
(a) Data collection method	Cross-sectional survey	Focus Group Discussions (FGDs)

(b) Sampling	A 2-stage stratified cluster sampling was employed, i.e., 20 union councils (smallest administrative structure of Pakistan) were selected as the primary sampling unit and then 25 households in each union council were marked as the secondary sampling unit (with an interval of 2 houses for urban clusters and 1 for rural clusters). One adult family member of each household was randomly selected by the Kish technique and interviewed. In case the selected member was not available, the team would try to locate him/her or else move on to the next household. Minors and acutely sick members of the family were excluded.	Purposive sampling was used to include the specific participants for FGDs.

(c) Sample size	The total sample comprised 526 individuals for which the following formula was used: *n* = Z_2*α*_(*P*) × (1 − *P*)/*d*^2^ (ignoring the design effects) where “*d*” is the difference between upper and lower limits of the interval estimate that is 5% (0.05) which is standard, “*p*” is prevalence, i.e., the probability of the indicator to be measured, and “*n*” is the sample size. By custom, one wants 95% confidence (*Z* ≥ 1.962) that the true value for an indicator would be within two standard errors of prevalence (*p*). The confidence level is assumed 95% precision, 5% point (*d* = 0.05), and conventionally, the “*p*” value is assumed 50% to work out the maximum sample size, in such situations where no prior estimates of “*p*” are available from any secondary sources. Hence, n = 196.2 (upward rounded to 197), and then using a design effect of 2 (197 x 2 = 394), and an expected response rate 75% (394/0.75 = 525.33), the final sample size was rounded off to 526.	Five FGDs were conducted with 47 participants in all. Each FGD comprised 8-12 participants and lasted from 50 to 90 minutes. (i) District health authorities, i.e., District Health Officer, Manager TB program, and District Managers of other health programs. (ii) Lady health workers, NGO workers, and private health providers (iii) Opinion leaders, i.e., teachers, imam mosque, barber, shopkeepers, ex-service men, social workers, community activists, etc. (iv) Male TB patients under treatment (v) Female TB patients under treatment

(d) Data collection tool	Validated structured questionnaire	FGD guide with main questions, follow-up questions, and probes

(e) Data analysis	Data entered in MS Excel and analysed in SPSS v22 for computing simple descriptive frequencies and for seeing significant statistical association by applying Pearson's chi-squared test.	Transcripts manually coded. An analytical approach, i.e., framework analysis, was adopted, and then coding was done to identify patterns, subthemes, and themes.

## Data Availability

First author has all the data of the study which can be requested for further analysis and research.
